# Ixodidae ticks in sheep and cattle in the Basilicata region (southern Italy)

**DOI:** 10.1186/1756-3305-7-S1-P8

**Published:** 2014-04-01

**Authors:** L Rinaldi, ME Morgoglione, E Noviello, A Bosco, G Prestera, G Cringoli

**Affiliations:** 1 Department of Veterinary Medicine and Animal Productions, University of Naples Federico II, Regional Center for Monitoring Parasitic Infections (CREMOPAR, Regione Campania), Naples, Italy; 2 Regional Association of Farmers, Basilicata, Italy; 3 CIRPAR, Italy

## 

Ixodidae are an important health problem for domestic and wild animals for direct damages caused by these ticks, but mostly because they are involved in the transmission of many diseases (tick borne diseases - TBDs). Therefore, the surveys on the presence and geographical distribution of ticks in domestic ruminants need to be constantly updated.

The aim of the present study was to update the data on the presence and distribution of Ixodidae ticks in cattle and sheep bred in the Basilicata region (southern Italy), an area with a Mediterranean climate favourable to the biology and ecology of ticks. In this region, the breeding of pasturing cattle and sheep is a widespread reality very important from an economical point of view, especially for the natural vocation of animals in using marginal hilly and mountainous pastures.

From May to September 2013, sheep and cattle farms (n = 82 and 31, respectively) were visited and ticks were collected from 20 animals per farm, preserved in ethanol 70% and then identified at species level using the morphometric keys present in the literature.

A total of 2,179 ticks were collected. The following species were identified in the sheep farms (total = 1594 adult ticks collected): *Rhipicephalus bursa *(67.1%), *R. sanguineus *(34.1%), *R. turanicus *(25.6%), *Dermacentor marginatus *(6.1%), *Hyalomma detritum *(3.7%), *Ixodes ricinus *(3.7%) and *Hyalomma marginatum *(1.2%). In the cattle farms, a total of 585 adult ticks were collected and the following species identified: *R. bursa *(61.3%), *H.marginatum *(29.0%), *R. turanicus *(25.8%) *sanguineus *(25.8%), *D. marginatus *(9.7%), *I. ricinus *(9.7%), *Ixodes gibbosus *(6.5%) and *Hyalomma detritum *(3.2%).

The results of the present cross-sectional survey confirm that the environmental and climatic conditions of southern Italy are suitable for different tick species infecting domestic ruminants that are therefore exposed to different TBDs.

